# THE UPSIDE OF GETTING OLD: TESTING A MODEL OF OLDER AGE AND BETTER EMOTIONAL WELL-BEING

**DOI:** 10.1093/geroni/igz038.2652

**Published:** 2019-11-08

**Authors:** Jenna Wilson, JoNell Strough, Natalie Shook

**Affiliations:** West Virginia University, Morgantown, West Virginia, United States

## Abstract

Older adults often report better emotional well-being than younger adults. Socioemotional Selectivity Theory posits that with age, reduced future time perspective motivates prioritizing emotional well-being in the present moment (Carstensen, 2006). Mindfulness, a receptive attention to experiences as they occur (Brown & Ryan, 2003), and savoring, the ability to regulate positive feelings in the moment (Bryant, 2003), are present-oriented processes associated with greater well-being. Recent evidence indicates that greater mindfulness in part accounts for age differences in positive affect (Shook et al., 2017). The current study investigated whether older age is associated with a greater present-oriented time perspective, which in turn is related to greater savoring and mindfulness, thus statistically accounting for older adults’ better well-being. Participants (N = 888, 20-88 years, Mage = 46.37, SD = 15.20) recruited via MTurk completed an online survey. Data were analyzed using structural equation modeling. The model provided an adequate fit to the data (CMIN/DF = 2.94, CFI = 0.985, RMSEA = 0.048). Older age was associated with greater present-oriented time perspective, and present-oriented time perspective was associated with greater savoring and mindfulness which, in turn, were associated with better emotional well-being. Alternative models were tested, but did not significantly improve model fit. Findings suggest that there may be benefits for younger adults’ well-being if they learned to be more present focused, savor the moment, and be more mindful like older adults. Thus, present-oriented time perspective may be an important factor for healthy aging.

